# Insight Into the Molecular Mechanisms Underpinning the Mycoremediation of Multiple Metals by Proteomic Technique

**DOI:** 10.3389/fmicb.2022.872576

**Published:** 2022-06-03

**Authors:** Priyadarshini Dey, Anushree Malik, Dileep Kumar Singh, Sven-Bastiaan Haange, Martin von Bergen, Nico Jehmlich

**Affiliations:** ^1^Applied Microbiology Lab, Centre for Rural Development and Technology, Indian Institute of Technology Delhi, New Delhi, India; ^2^Department of Molecular Systems Biology, Helmholtz Centre for Environmental Research, Helmholtz Association of German Research Centres (HZ), Leipzig, Germany; ^3^Department of Biotechnology, MS Ramaiah Institute of Technology, Bengaluru, India; ^4^Department of Zoology, Faculty of Science, University of Delhi, New Delhi, India; ^5^Institute of Biochemistry, Faculty of Biosciences, Pharmacy and Psychology, University of Leipzig, Leipzig, Germany; ^6^German Centre for Integrative Biodiversity, Research (iDiv) Halle-Jena-Leipzig, Leipzig, Germany

**Keywords:** multi-metal, proteomics, mycoremediation, LC-MS/MS, fungi

## Abstract

We investigated the fungus *Aspergillus fumigatus* PD-18 responses when subjected to the multimetal combination (Total Cr, Cd^2+^, Cu^2+^, Ni^2+^, Pb^2+^, and Zn^2+^) in synthetic composite media. To understand how multimetal stress impacts fungal cells at the molecular level, the cellular response of *A. fumigatus* PD-18 to 30 mg/L multimetal stress (5 mg/L of each heavy metal) was determined by proteomics. The comparative fungal proteomics displayed the remarkable inherent intracellular and extracellular mechanism of metal resistance and tolerance potential of *A. fumigatus* PD-18. This study reported 2,238 proteins of which 434 proteins were exclusively expressed in multimetal extracts. The most predominant functional class expressed was for cellular processing and signaling. The type of proteins and the number of proteins that were upregulated due to various stress tolerance mechanisms were post-translational modification, protein turnover, and chaperones (42); translation, ribosomal structure, and biogenesis (60); and intracellular trafficking, secretion, and vesicular transport (18). In addition, free radical scavenging antioxidant proteins, such as superoxide dismutase, were upregulated upto 3.45-fold and transporter systems, such as protein transport (SEC31), upto 3.31-fold to combat the oxidative stress caused by the multiple metals. Also, protein–protein interaction network analysis revealed that cytochrome c oxidase and 60S ribosomal protein played key roles to detoxify the multimetal. To the best of our knowledge, this study of *A. fumigatus* PD-18 provides valuable insights toward the growing research in comprehending the metal microbe interactions in the presence of multimetal. This will facilitate in development of novel molecular markers for contaminant bioremediation.

## Graphical Abstract

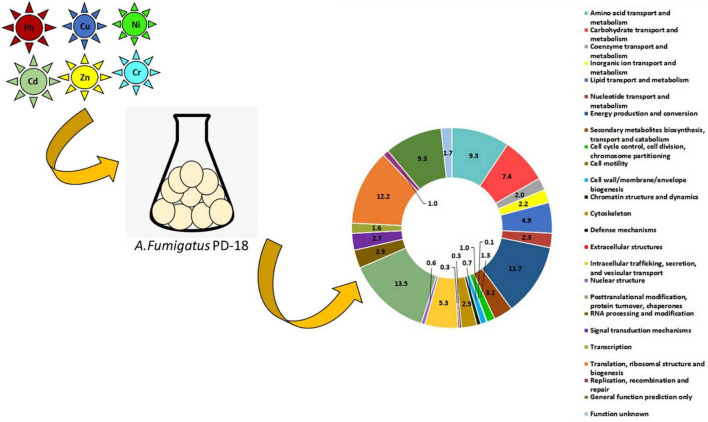

## Highlights

- First proteomic study on filamentous fungus *Aspergillus fumigatus* for hexametal removal.
- Most upregulated class posttranslational modification, protein turnover, chaperones.
- Major protein player in protein-protein interaction identified as cytochrome oxidase.
- Expressed proteins are environmental biomarkers in heavy metals-fungus interaction.

## Introduction

Heavy metals, such as copper (Cu), chromium (Cr), cadmium (Cd), zinc (Zn), lead (Pb), and nickel (Ni), occur in the water bodies, such as river water, and drains, in developing countries that are above the permissible mandates as prescribed by the Food and Agriculture Organization (Bhattacharya et al., 2015). These heavy metals are released from numerous small- and medium-scale enterprises (pesticide, textile, electroplating, fertilizer, batteries, etc.) due to lack of or improper sewage treatment plant systems (Bhattacharya et al., 2015; Bhardwaj et al., 2017; Gola et al., 2020). These hazardous contaminants are discharged into various water bodies *via* irrigation during agricultural activities. This results in bioaccumulation of these contaminants that enter the food chain and cause detrimental health ailments, such as cancer in human beings (Kou et al., 2018; Li et al., 2019; Abed et al., 2020; Chauhan et al., 2021; Irawati et al., 2021). Thus, these heavy metals are the environmental priority contaminants threatening the environment and therefore must be remediated before discharge into the environment.

The conventional physico-chemical methods, such as chemical precipitation, ion exchange, adsorption, membrane filtration, coagulation-flocculation, and flotation, are usually utilized to remediate these harmful contaminants. These techniques have the disadvantages of being expensive, having low selectivity, production of additional sludge, and further treatment is required for better results (Dey et al., 2021; Zamora-Ledezma et al., 2021).

On the other hand, living and actively growing microbial cells can be a lucrative option for bioremediation (Malik, 2004). Of the bioremediation techniques, mycoremediation has shown to be a promising technology having the potential to ameliorate these hazardous chemicals (Dey et al., 2020; Sabuda et al., 2020). The superiority of fungi over single-celled microbes, such as bacteria, to remediate these recalcitrant heavy metals is well-documented (Deshmukh et al., 2016). In addition, the fungi are omnipresent, multifarious, and have a wider arsenal to acclimatize to environmental limitations, such as immoderations of temperature, extremes of pH, higher metal concentrations, and low nutrient accessibility, due to their morphological diversity (Anand et al., 2006). Besides, fungal mycelia have enhanced enzymatic and mechanical contact with the pollutant due to a greater cell to the surface ratio (Sagar and Singh, 2011).

Further, in microbial cells, such as fungus, heavy metals are key components in the number of catalytic and structural proteins that are integral to biochemical processes. The outcome of these processes differs depending on the type of metal involved and its concentration inside the cell (Gadd, 1994). Particularly, the species of *Aspergillus* have a high metal uptake capacity for metals, such as Cu (Dusengemungu et al., 2020). Also, filamentous fungi develop signature metabolic pathways which are species-specific to survival in the harsh environment of heavy metals and other contaminants that are utilized as nutrients and energy sources (Kuhn and Käufer, 2003). Accordingly, proteins expressed in cells under such diverse conditions and at different times are dissimilar (Boopathy, 2000; Keller, 2015; Wisecaver and Rokas, 2015).

Also, there is evidence that fungal resistance toward one element does not necessarily infer resistance to another element even though the elements possess similar valency charges (Høiland, 1995). For example, the toxicity of Pb toward the microbe is less compared to other toxic metals, such as Cd, As, and Hg (Jiang et al., 2020). Further, in fungi, the metallothioneins formation is predominantly induced by the heavy metal Cu (Jaeckel et al., 2005). Thus, understanding the altered heavy metal uptake in the fungus in presence of diverse environmental conditions needs to be evaluated.

To gain such mechanistic insight, high throughput techniques, such as proteomic analysis by LC-MS/MS, can be used to identify and characterize proteins involved in the multimetal resistance mechanism in a filamentous fungus (Pandey and Mann, 2000; Kraut et al., 2009; Zhang et al., 2010). Moreover, proteomics facilitates the development of new and important protein biomarkers that specify and monitor metal contamination in the environments as protein type and also their copy numbers are estimated by the translational regulation (Ohno et al., 2014).

Several researchers have studied the change in proteomes under various metal conditions. Selamoglu et al. (2020) have assessed the change in the differential proteome expression of the prokaryotic organism *Rhodobacter capsulatus* in presence of 5 μM Cu by nano-LC-MS/MS. About 75 proteins were significantly regulated. Most of the proteins present were responsible for maintaining Cu homeostasis. Further, Lotlikar et al. (2020) enumerated the variable expression of proteins in a eukaryotic fungus *Penicillium chrysogenum* in the presence of 100 mg/L and 500 mg/L Cu. Several key proteins related to genetic information, carbohydrate metabolism, glycan biosynthesis and metabolism, amino acid metabolism, and energy metabolism were expressed.

Cherrad et al. (2012) highlighted the overaccumulation of proteins of the oxidoreductase family when exposed to Cd, Cu, and Ni but not when exposed to Zn. Thus, the secretion of proteins in fungus is extremely dynamic and its production depends on different environmental triggers.

However, despite these growing proteomic studies dealing with the bioremediation of single heavy metal, there is a dearth of information on the modulated proteins triggered by the cumulative toxicity of hexametals, namely, Cd, Cr, Cu, Ni, Pb, and Zn, specifically in a filamentous fungus. Interestingly, *Aspergillus fumigatus* has high adaptability when subjected to an altered environment (Bakti et al., 2018). Also, as established in our previous works (Dey et al., 2016, 2020; Bhattacharya et al., 2020), *A. fumigatus* PD-18 is a filamentous fungus capable of removal of 30 mg/L multimetal exceptionally well. Furthermore, there are structural and functional similarities between the numerous genes of lower eukaryotes, such as fungi and mammals (Bae and Chen, 2004).

Thus, this fungus *A. fumigatus* PD-18 would be a good eukaryotic model for helping us understand how cells adopt various cellular strategies and lay a foundation study to decipher the enzymes produced in the presence of simultaneous effects of multimetal cocktail on interaction with a fungus for scale-up process.

## Materials and Methods

### Chemicals and Reagents

The stock solutions (10 g/L) of different individual metals were made by dissolving their respective salts, viz. K_2_Cr_2_O_7_, Cd(NO_3_)_2_, Ni(NO_3_)_2_, Cu(NO_3_)_2_, Zn(NO_3_)_2_, and Pb(CH_3_COO)_2_ in double-distilled water and were diluted to the concentrations that were required for the experiments. For preparing the reagents and calibration standards, deionized ultrapure water (RIONS Ultra 370 series) was used. Rest all other chemicals utilized were of analytical grade and were obtained from Merck, Sigma, and Qualigens.

### Microorganism and Culture Media Composition

The fungal strain used was *A. fumigatus* PD-18 which was isolated from the polluted banks of the river Yamuna, New Delhi, India, and characterized with accession number KX365202 after depositing the sequence to the Genbank (NCBI) (Dey et al., 2016).

The growth media used was the composite medium with following composition (g/L): (NH_4_NO_3_, 0.5; K_2_HPO_4_, 0.5; MgSO_4_.7H_2_O, 0.1; NaCl, 1.0; Yeast extract, 2.5; pH 6.8 ± 0.2). The sterilization of the media was carried out at 121°C for 15 min. Glucose (10.0 g/L) was added to the flasks separately after autoclaving to avoid precipitation.

### Methodology

The methodology adopted in this study was to estimate the differential expression of proteins in *A. fumigatus* PD18 brought about by 30 mg/L multimetal (MM) viz. Five milligram per liter of each of the individual Cd, Total Cr, Cu, Ni, Pb, and Zn amended in the composite media in addition to 1% of glucose. The concentration of 5 mg/L of each heavy metal was chosen to take into consideration the permissible mandates for heavy metal occurrence in water that can be utilized for irrigation according to Food and Agriculture Organization (FAO). The prescribed limit for each heavy metal is as follows: Cd, 0.01; Total Cr, 0.1; Cu, 0.2; Ni, 0.2; Pb, 5.0; Zn, 2.0. Also, the typical concentration of heavy metals occurring in a mixture in the Yamuna river was considered (Bhattacharya et al., 2015). The biotic control included composite media added with only 1% of glucose.

Studies were conducted in a series of Erlenmeyer flasks (250 mL) comprising 100 mL of composite growth media. One milliliter of spore suspension (having a concentration of 10^7^ spores eluted with sterile distilled water containing 0.01% Tween 80) was inoculated in the flasks and incubated at 30°C and 150 rpm agitation for 72 h to ensure complete uptake of metal ions. The flasks were withdrawn after the late log phase incubation period. Analysis was done in three technical replicates for biotic control and multimetal treated samples separately.

### Proteomics Analysis

#### Cell Lysis and Protein Extraction

The fungal pellets were separated from the media by inversion and followed by centrifugation at 4,000 g for 10 min at 4°C. The mycelia were further snap-freezed in liquid nitrogen and then lyophilized and stored as a dry powder at –20°C before the subsequent steps. The total protein from the dried powdered fungal cells was extracted in a buffer of composition (8M Urea and 2M Thiourea) as per the modified protocol (Rughöft et al., 2020). The supernatant was collected by centrifugation at 14,000 rpm, 4°C for 10 min, and precipitated overnight in five volumes of ice-cold acetone and the pellet was stored at –20°C for further use. The concentration of protein to be measured was estimated according to the Bradford assay of protein quantification (Bradford, 1976). In brief, a 2-mg/mL concentration stock solution of Bovine Serum Albumin (BSA) was prepared in water. BSA standard curve was prepared using the following concentration range (0–2.00 μg/μL) followed by the addition of Coomassie Brilliant Blue dye reagent in 95% ethanol and 100 mL 85% (w/v) phosphoric acid and incubated for 5 min. The absorbance was measured at 595 nm.

#### Protein Separation by 1D-SDS PAGE and in Gel Tryptic Digestion of the Proteins

The separation of the proteins was carried out by one-dimensional SDS-PAGE and the separated protein bands were visualized by Coomassie brilliant blue staining. The individual gel bands were cut into small pieces and washed with 10 mM ammonium bicarbonate followed by reduction with 10 mM 1,4-Dithiothreitol and 100 mM 2-Iodoacetamide. The proteins lysates were subjected to tryptic digestion and incubated overnight and kept at 37°C. The peptides were extracted from the gel pieces in 5 mM ammonium bicarbonate and further purified by C-18 ZipTip columns according to the protocol (Haange et al., 2012). Thereafter, the peptide lysates were dried in a vacuum centrifuge and stored at –20°C until further analysis.

### Mass Spectrometric Analysis

The dried peptides lysates were reconstituted in 0.1% (v/v) formic acid and loaded and separated by reversed-phase chromatography before being measured on mass spectrometer UPLC-coupled (Waters) LTQ Orbitrap Velos MS/MS (Thermo Fisher Scientific).

### Data Analysis (Identification and Characterization of the Proteins)

The raw MS-MS ion spectra data from the instrument were processed using the Proteome Discoverer software (v1.0 build 43, Thermo Fisher Scientific) (Orsburn, 2021). The fixed and variable modifications (Carbamidomethylation at cysteines was given as fixed and oxidation of methionines as variable modifications) were taken into consideration. Only ranked one peptide hits with ≥1 high confidence and less than 1% false discovery rate (FDR) were considered as identified and taken up for further analysis. The intensity data were further processed by logarithmic transformation and normalization for the determination of changes in the abundance of proteins (at least 1.5-fold upregulated or downregulated). Only the values of *p* < 0.05 were considered as statistically significant. Triplicate gels were run for biotic control and MM samples. For functional annotation of the proteins, the generated protein lists were analyzed by prophane.^1^

The creation of the VENN diagrams was done by the online tool VENNY.^2^ The heatmaps and volcano plots were constructed using the heatmap and volcano programs from R tool version 3.2.0. The WoLF PSORT tool^3^ was used for the prediction of the subcellular localization of the proteins. The protein–protein interactions between the differentially expressed proteins were studied by the STRING (Search Tool for the Retrieval of Interacting Genes/Proteins) database.^4^

## Results

Fungi usually acclimate by making dynamic changes in the cell structure and composition when exposed to heavy metals. In our previous study on the toxicity of individual metals, that is, Cd, Cr, Cu, Ni, Pb, and Zn (500 mg/L) in *A. fumigatus* PD-18, we determined the highest tolerance index for each metal in solid media. We calculated the cube root growth (k) constant of *A. fumigatus* PD-18 when subjected to 30 mg/L multimetal in liquid composite media. This fungal strain had exceptional multimetal removal ability after ∼72 h. The growth profile of the fungus was altered when multimetal was added to the composite media and substantial variations were observed when compared with individual metals (Dey et al., 2016).

Further, the morphological changes in this fungus in response to the 30 mg/L multimetal were determined by scanning electron microscope, the localization of the heavy metals inside the fungal cell by transmission electron microscopy, and chelation of the heavy metals with the functional groups occurring in the fungus by Fourier-transform infrared spectroscopy (Dey et al., 2020).

### Expression of Proteins in Biotic Control and Multimetal Extracts of *Aspergillus fumigatus* PD-18

From the six sample preparations (conditions, including one biological control and one multimetal exposure and their three technical replicates), 434 proteins were uniquely present in multimetal extracts and 400 proteins in biotic control of *A. fumigatus* PD-18.

**FIGURE 1:**
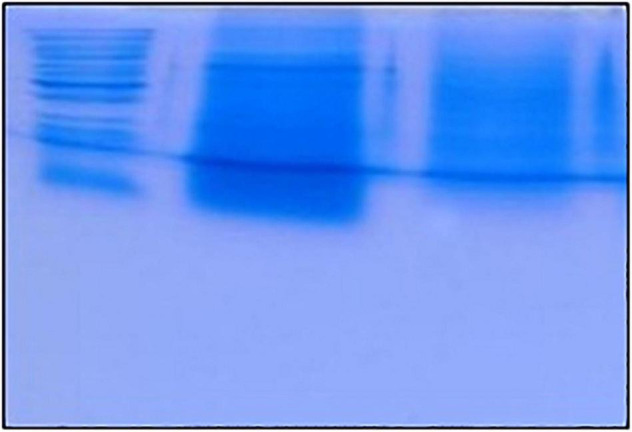
Coomassie brilliant blue stained segment of SDS-PAGE protein expression patterns in heavy metal treated *Aspergillus fumigatus* in absence of multimetal (Biotic control in 2^nd^ lane); 30 mg/L multimetal (3^rd^ lane). Molecular-weight markers (M) are shown on the left (1^st^ lane).

Figure 1 depicts the expression of the proteins isolated from *A. fumigatus* PD-18 mycelia as established with SDS-PAGE. This expression of proteins from the biotic control and multimetal (MM) stressed PD-18 were observed after Coomassie Brilliant Blue staining.

### Proteins Present in High Abundances in *Aspergillus fumigatus* PD-18

**FIGURE 2:**
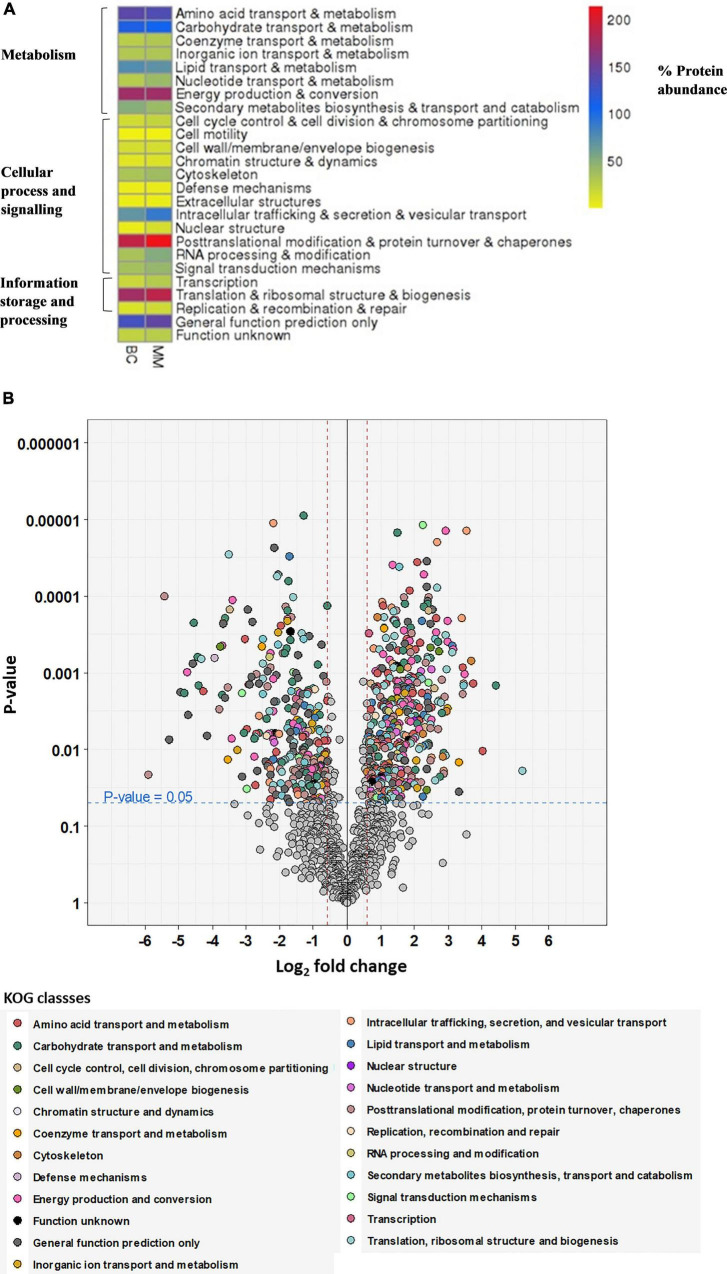
**(A)** Heatmap visualization of the abundance of the proteins as red = higher values, dark blue = mid values, yellow = lower values depicted on (x-axis) for proteome abundance of 30 mg/L multimetal (MM) and proteome abundance without heavy metal exposure (BC). **(B)** Volcano plot showing the distribution of quantified proteins according to *p-*value and log_2_ fold change, indicating significance level at 0.05 (blue dashed line) and fold change at –1.5 and 1.5 (red dashed lines), colored according to their KOG class. Proteins, which were not significant or fold change within –1.5 to 1.5 are colored in gray.

Figure 2A depicts the heatmap of differently regulated proteins of the multimetal exposure when compared to the biotic control without multimetal. The detected proteins were categorized under three functional KOG groups related to metabolism, cellular process and signaling, and information storage and processing. These categories were associated with fungal physiology and most of the identified proteins were further classified on the basis of different 23 KOG classes which are discussed as follows:

• metabolism [amino acid transport and metabolism (9%), carbohydrate transport and metabolism (7%), coenzyme transport and metabolism (2%), inorganic ion transport and metabolism (2%), lipid transport and metabolism (5%), nucleotide transport and metabolism (3%), energy production and conversion (11%), and secondary metabolites biosynthesis and transport and catabolism (3%)];
• cellular process and signaling [cell cycle control and cell division and chromosome partitioning (1%), cell motility (0.1%), cell wall/membrane/envelope biogenesis (1%), chromatin structure and dynamics (1%), cytoskeleton (3%), defense mechanisms (0.3%), extracellular structures (0.3%), intracellular trafficking, secretion, and vesicular transport (6%), nuclear structure (1%), post-translation modification and protein turnover and chaperones (14%), RNA processing and modification (3%), and signal transduction mechanism (3%)];
• information storage and processing [transcription (2%), translation and ribosomal structure and biogenesis (12%), replication, recombination, and repair (1%), proteins with general function (9%), and function unknown (2%)].

From Figure 2A, the proteins that were expressed in higher abundance in *A. fumigatus* when treated with 30 mg/L multimetal belonged to the main metabolic functional classes of RNA processing & modification, post-translational modification & protein turnover & chaperones, nuclear structure, intracellular trafficking & secretion & vesicular transport, translation & ribosomal structure & biogenesis, and nucleotide transport & metabolism. Further, hypothetical proteins with general function prediction were also expressed which could play role in tackling the multimetal stress. The afflicted metabolic functions classes where production of proteins decreased belonged to amino acid transport & metabolism, carbohydrate transport & metabolism, lipid transport & metabolism, and secondary metabolites biosynthesis & transport and catabolism. This proteomic study displayed proteins in *A. fumigatus* PD-18 that showed a significant fold increase and decrease of differentially expressed proteins during growth in 30 mg/L multimetal supplemented composite media as depicted in the volcano plot in Figure 2B. Among the differentially expressed proteins identified, 399 proteins were differentially upregulated while 266 proteins were differentially downregulated. The highly upregulated protein identified hydroxymethylglutaryl-CoA synthase upregulated upto 5.2-fold times. Other important proteins with general function prediction were upregulation of serine/threonine-protein phosphatase upto 3.42-fold times and G-protein beta subunit SfaD upto 2.98-fold. The highly upregulated and downregulated proteins by fold changes and their locations inside the fungal cell are depicted in Table 1. Furthermore, it was found that the majority of the upregulated proteins were located in the cytoplasm (18), mitochondria (12) followed by extracellular (6), nuclear (5), plasma membrane (2), peroxisome (2), nuclear and cytoplasmic (1), and endoplasmic reticulum lumen (1).

**TABLE 1:** Differential abundance of highly regulated selected proteins in *Aspergillus fumigatus* grown in presence 30 mg/L MM.

Protein accession number	Protein name	Fold change	Cellular location of protein	Protein KOG class	*p*-value
**Upregulated proteins**					
A1CQV3	hydroxymethylglutaryl-CoA synthase	5.19	Cytoplasm	Lipid transport and metabolism	0.019
B0XZB6	aminotransferase family protein	4.03	Mitochondria	Amino acid transport and metabolism	0.010
Q4WV94	proteasome regulatory particle subunit Rpt3	3.74	Peroxisome	Post-translational modification, protein turnover, chaperones	0.001
Q4WXX9	fibrillarin	3.68	Cytoplasm	RNA processing and modification	0.001
Q4X0E9	glutamate dehydrogenase	3.54	Mitochondria	Amino acid transport and metabolism	0.001
Q4WLA8	superoxide dismutase	3.45	Mitochondria	Inorganic ion transport and metabolism	0.001
A0A017SPG0	calcium/calmodulin-dependent protein kinase	3.45	Cytoplasm	Signal transduction mechanisms	0.001
A0A017SD45	ATP synthase	3.44	Mitochondria	Energy production and conversion	0.001
Q4WM81	serine/threonine-protein phosphatase	3.42	Nuclear	Signal transduction mechanisms	0.001
A1C6S1	histone H4	3.40	Mitochondria	Chromatin structure and dynamics	0.001
A0A084BYE7	protein transport protein (SEC31)	3.31	Mitochondria	Intracellular trafficking, secretion, and vesicular transport	0.036
A0A017S654	glutamate decarboxylase	3.16	Peroxisome	Amino acid transport and metabolism	0.001
A2R0I8	26S protease regulatory subunit	3.12	Nuclear and cytoplasmic	Post-translational modification, protein turnover, chaperones	0.001
B0XVU3	RNA binding effector protein Scp160	3.11	Endoplasmic reticulum lumen	Lipid transport and metabolism	0.001
O60022	probable HECT-type ubiquitin ligase	2.99	Extracellular	General function prediction only	0.002
A1CI51	G protein beta subunit SfaD	2.98	Cytoplasm	General function prediction only	0.001
Q4WLV1	26S proteasome regulatory particle subunit Rpn8	2.96	Extracellular	Post-translational modification, protein turnover, chaperones	0.001
Q4WFD3	coatomer subunit gamma	2.94	Cytoplasm	Intracellular trafficking, secretion, and vesicular transport	0.012
A0A017S6B4	phosphoglycerate kinase	2.91	Mitochondria	Carbohydrate transport and metabolism	0.001
Q4WX43	endosomal cargo receptor (P24)	2.86	Nucleus	Intracellular trafficking, secretion, and vesicular transport	0.020
A0A084C1K3	glutamyl-tRNA synthetase	2.86	Nucleus	Translation, ribosomal structure and biogenesis	0.011
A0A017S4K9	pyridoxine biosynthesis protein	2.82	Cytoplasm	Coenzyme transport and metabolism	0.001
B0XXM2	CBS and PB1 domain protein	2.73	Extracellular	Energy production and conversion	0.002
A1CPB0	sulfate adenylyltransferase	2.71	Cytoplasm	Inorganic ion transport and metabolism	0.001
A0A084BD28	importin beta 4 subunit	2.69	Nucleus	Nuclear structure	0.002
Q4WHV3	oxidoreductase short chain dehydrogenase	2.58	Plasma membrane	Secondary metabolites biosynthesis, transport and catabolism	0.001
Q4 × 0N5	T-complex protein 1 subunit gamma	2.5	Nucleus	Post-translational modification, protein turnover, chaperones	0.012
A0A084BP78	acetolactate synthase	2.49	Mitochondria	Amino acid transport and metabolism	0.005
Q0CSN0	Mitochondrial inner membrane translocase subunit TIM44	2.41	Mitochondria	Intracellular trafficking, secretion, and vesicular transport	0.002
Q4WE44	Cytochrome P450 phenylacetate 2-hydroxylase	2.39	Cytoplasm	Secondary metabolites biosynthesis, transport and catabolism	0.001
I7ZRN7	glyceraldehyde-3-phosphate dehydrogenase	2.38	Cytoplasm	Carbohydrate transport and metabolism	0.001
A4FSH5	RAB proteins geranylgeranyltransferase component A	2.37	Extracellular	Post-translational modification, protein turnover, chaperones	0.001
Q5B2V1	Peptidyl-prolyl *cis*-trans isomerase D	2.37	Cytoplasm	Post-translational modification, protein turnover, chaperones	0.001
B8NQD2	mitochondrial enoyl reductase	2.35	Cytoplasm	Transcription	0.033
Q4WGJ9	Tropomyosin	2.26	Mitochondria	Cytoskeleton	0.041
Q4WIT8	Ubiquitin DskB	2.13	Extracellular	Post-translational modification, protein turnover, chaperones	0.004
A0A084BHN1	Importin beta 5 subunit	2.02	Cytoskeleton	Intracellular trafficking, secretion, and vesicular transport	0.018
** Upregulated proteins**					
Q4WJB0	ribonucleoside-diphosphate reductase	1.93	Mitochondria	Nucleotide transport and metabolism	0.001
Q4W9K3	Mitochondrial outer membrane translocase receptor (TOM70)	1.84	Extracellular	Intracellular trafficking, secretion, and vesicular transport	0.001
G7XPM0	ATP-dependent bile acid permease	1.72	Cytoplasm	Secondary metabolites biosynthesis, transport and catabolism	0.014
Q4 × 237	translation release factor eRF3	1.71	Mitochondria	Translation, ribosomal structure and biogenesis	0.002
Q4WVZ4	isocitrate dehydrogenase LysB	1.69	Nucleus	Amino acid transport and metabolism	0.013
Q96UX3	14-alpha sterol demethylase	1.69	Cytoplasmic and nuclear	Secondary metabolites biosynthesis, transport and catabolism	0.001
A0A084C1V0	DNA damage inducible v SNARE binding protein Ddi1	1.65	Cytoplasm	Replication, recombination and repair	0.018
Q5B7M1	Vesicular-fusion protein sec17	1.65	Cytoplasm	Intracellular trafficking, secretion, and vesicular transport	0.001
A0A084BWQ6	Nuclear pore complex subunit (SEC13)	1.62	Cytoplasmic and nuclear	Intracellular trafficking, secretion, and vesicular transport	0.001
Q0CCZ9	bifunctional purine biosynthetic protein Ade1	1.60	Cytoplasm	Nucleotide transport and metabolism	0.020
A0A017SJP6	protein phosphatase 2a 65kd regulatory subunit	1.54	Plasma membrane	Signal transduction mechanisms	0.044
Q4WN17	septin	1.54	Cytoplasm	Cell cycle control, cell division, chromosome partitioning	0.001
A1CB85	transcription elongation factor spt4	1.49	Cytoplasm	Post-translational modification, protein turnover, chaperones	0.001
P20445	MICOS complex subunit mic60	1.39	Cytoplasm	Cell wall/membrane/envelope biogenesis	0.003
Q6MYF0	plasma membrane H +-ATPase Pma1	1.28	Cytoplasm	Inorganic ion transport and metabolism	0.005
**Downregulated proteins**					
Q4WHK1	Small nuclear ribonucleoprotein Sm D1	−0.59	Nucleus	Translation, ribosomal structure, and biogenesis	0.034
Q4WHE2	NADH-ubiquinone oxidoreductase	−0.59	Cytoplasm	Energy production and conversion	0.002
B0Y5W4	Pyruvate carboxylase	−0.61	Peroxisome	Post-translational modification, protein turnover, chaperones	0.029
G3XYJ1	GST C-terminal domain-containing protein	−0.63	Cytoplasm and nucleus	Amino acid transport and metabolism	0.011
Q4WCQ8	ATP synthase subunit E	−0.64	Nucleus	Energy production and conversion	0.012
Q4 × 0B7	Arginyl-tRNA synthetase	−0.68	Cytoplasm	Lipid transport and metabolism	0.012
A1CIL4	Histidine–tRNA ligase	−0.75	Mitochondria	Coenzyme transport and metabolism	0.029
B0XS44	Carbamoyl-phosphate synthase	−0.77	Mitochondria	Signal transduction mechanisms	0.013
Q6QNB5	Squalene monooxygenase	−0.78	Plasma membrane	Translation, ribosomal structure and biogenesis	0.010
Q4WY15	Arginase	−0.77	Mitochondria	Energy production and conversion	0.016

### Enriched Protein Network and Pathways in *Aspergillus fumigatus* PD-18

**FIGURE 3:**
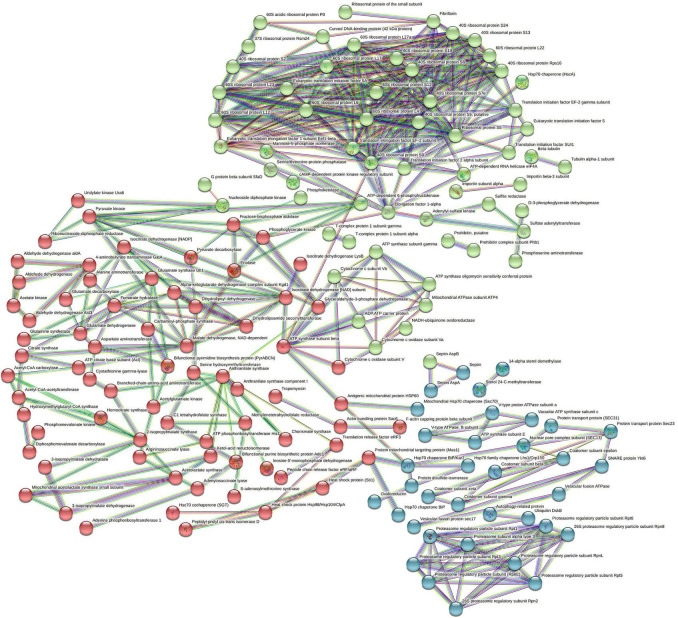
STRING analysis displaying protein–protein interaction. The colored lines represent the edges that show associations among different proteins. Dark blue: connect proteins that are co-occurring phylogenetically. Dark green: connect proteins that are occurring as gene neighborhood. Red: shows proteins with gene fusions. The colored nodes (red, blue, green) are different clusters of proteins.

The interaction network as constructed by the STRING database of the highly upregulated proteins in *A. fumigatus* is depicted in Figure 3. The strength of the association is depicted by the thickness of the line. The number of nodes is 263 that represents the proteins, the number of edges is 467 that represents associations, the average node degree is 3.55, and the average local clustering coefficient is 0.412. The STRING analysis identified three clusters of protein interactions. The first major cluster of proteins (green) was involved in ribosome and preinitiation factors (60S and 40S ribosomal proteins). The predicted partners functionally associated with 60S and 40S ribosomal proteins include translation elongation factor, fibrillarin of class translation, ribosomal structure, and biogenesis. The second cluster proteins (red) have major hub proteins, such as malate dehydrogenase, ATP synthase, pyruvate kinase, fructose biphosphate aldolase, dihydrolipoyl dehydrogenase, glutamate synthase, anthranilate synthase, and isocitrate dehydrogenase. The third cluster proteins (blue) are proteasome regulatory particles, Hsp70 chaperone, protein transport protein (sec31), and V-type proton ATPase.

**FIGURE 4:**
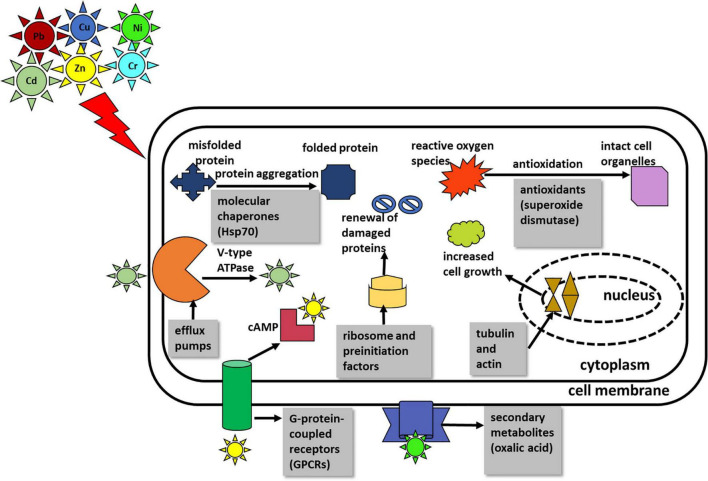
Summary of the salient mechanism of multimetal detoxification process by *Aspergillus fumigatus* PD-18.

The significant junction proteins in protein–protein interaction identified were cytochrome c, oxidase, and septin suggest that energy production and conversion and cell cycle control, cell division, and chromosome partitioning that play a pivotal role in the resistance toward multimetal exposure. Figure 4 summarizes the salient mechanism of the multimetal detoxification process by *A. fumigatus* PD-18.

## Discussion

In this study, we identified the chief classes of proteins that are crucial for the resistance and tolerance of *A. fumigatus* PD-18 for multimetals, including intracellular and extracellular mechanisms of metal uptake. These two processes are further elaborated.

### Intracellular and Extracellular Mechanism of Metal Uptake by Fungus

The mechanism of the fungal resistance and tolerance could be attributed to its occurrence at the metal-contaminated site (Baker, 1987; Gadd and White, 1993). The fungal detoxification process involves strategies, such as intracellular bioaccumulation, extracellular precipitation, biotransformation, biomineralization, and biosorption, that involve several signaling pathways (Wang et al., 2019). In addition, the reduction of heavy metals from the cell comprises of activation of different metabolic processes in the cell that are stimulated by heavy metals (Goyal et al., 2003).

Fungi largely counter heavy metals in two ways. The first way involves averting the metal uptake and its passage inside the fungal cell. This happens chiefly by a decrease in metal uptake or increased efflux of metals, metal biosorption to the impermeable cell walls by metal binding polysaccharides, peptides, extracellular formation of complexes, and the release of organic acids that chelates heavy metals outside the cell. Thus, the extracellular mechanism operates in the cell to counter heavy metals by circumventing the entry of heavy metals. Particularly, the secondary metabolites, such as citric acid, oxalic acids, succinate, and fumarate, which are low molecular weight compounds (<900 daltons) are secreted by fungal species namely *A. niger* specifically in response to heavy metals exposure and they bind the heavy metals extracellularly (Kolen, 2013). Also, the secondary metabolite oxalic acid is produced as an intermediate compound in the biochemical tricarboxylic acid cycle (TCA) (Munir et al., 2001). Here, we found the upregulation of the key enzymes that are involved in the TCA cycle viz. phosphoglycerate kinase (2.91-fold), glyceraldehyde-3-phosphate dehydrogenase (2.38-fold), enolase (1.8-fold), and pyruvate kinase (1.86-fold) which mediates the enhanced secretion of oxalic acids.

In the second way, the fungus subsists the high concentration of metals inside the cell by tolerance after the process of detoxification *via* metal chelation by synthesizing ligands, such as metallothioneins and phytochelatins, that bind heavy metals intracellularly or by compartmentalization of heavy metals within the cell organelles of vacuoles by polyphosphates. The three main classes of intracellular peptides binding metal ions are phytochelatins (PCs), metallothioneins (MTs), and glutathione (GSH). MTs are low molecular weight cysteine-rich metal-binding proteins that have high affinity toward both the essential metal ions, such as Cu and Zn, non-essential metal ions, such as Cd, Hg, and Ag, and also have large metal-binding capacities (Reddy et al., 2014). Further, MTs chelate heavy metals by forming thiolate bonds with the heavy metals. Glutathione S transferases (GSTs) are enzymes that metabolize heavy metals and other contaminants by catalyzing the binding of glutathione to non-polar compounds comprising of electrophilic nitrogen, carbon, and sulfur atom (Morel et al., 2009). Usually, the metals Cd, Cu, Pb, and Zn are removed *via* glutathione (GSH)-mediated sequestration. However, in this study, there was no evidence of the production of proteins glutathione, metallothioneins, and phytochelatins despite the presence of these heavy metals Cd, Cu, Pb, and Zn in the multimetal mixture. The reason for this phenomenon could be attributed to the dynamics of individual heavy metals when present in a mixture. As the expression of GST is related to the type of heavy metal, its concentration, and the extent of treatment time of the heavy metal (Shen et al., 2015).

Further, it is reported that heavy metals induce oxidative damage to the cell membranes of fungi by the generation of reactive oxygen species (ROS). These ROS are detoxified by the production of antioxidants that are components from the thioredoxin system, such as peroxiredoxins, NADPH dehydrogenases, catalase, superoxide dismutase, and peroxidase, that enables the fungus to confront the reactive-oxygen species that accumulate in the cell on exposure to the metals (Zhang et al., 2015). Thus, in principle, intracellular mechanisms decrease the metal load in the cytosol (Sandau et al., 1996; Gadd, 2000, 2007, 2010). In this study, we found 3.45-fold upregulation of the antioxidant protein Cu-Zn superoxide dismutase. Other important functional groups were detected that expressed amino acid metabolism, lipid metabolism, energy metabolism, and also the proteins involved in signal transduction, transcription, translation, or DNA repair. In general, the upregulated proteins are stimulated to display the fungal resistance against the contaminants’ stress, while the downregulated proteins are suppressed by the action of the pollutants’ toxicity.

The highly upregulated protein hydroxymethylglutaryl-CoA synthase with 5.19-fold upregulation is responsible for the production of secondary metabolite carotenoid from the precursor molecule of acetyl-CoA when stimulated by the heavy metal stress (Bhosale, 2004). The protein serine/threonine phosphatase with 3.42-fold upregulation is responsible for maintaining the conformation of cell organelles and proteasomes (Dias et al., 2019).

The other proteins, such as G-protein beta subunit *SfaD*, with 2.98-fold upregulation depicted the role of G-protein-coupled receptors (GPCRs) in heavy metal bioremediation. These are the largest transmembrane receptors that aid in communicating the extracellular signals, such as stresses of heavy metals into the intracellular sites. G-protein-coupled receptors (GPCRs) regulate the important effector molecules, such as adenylate cyclase and phospholipase C, and regulate the function of kinase and ion channel by producing secondary messengers, such as cAMP, thereby inducing signaling cascades (El-Defrawy and Hesham, 2020).

From Figures 2A, discussion of the highly regulated selected KOG classes are as follows:

#### Post-translational Modification, Protein Turnover, Chaperones

Protein homeostasis is crucial for the cell proliferation and viability of all organisms. Further, cellular signaling is greatly affected by the protein homeostasis under different physiological conditions and environmental stresses, such as heavy metals, and therefore they can be suitable biomarkers. Molecular chaperones aid in the delivery of metal ions to the cell organelles and metalloproteins (Lotlikar and Damare, 2018). The two vital regulators of molecular chaperones in the proteostasis network are heat shock transcription factor Hsf1 and heat shock protein Hsp90. Under a stressful environment, heat shock protein enables the folding of newly synthesized proteins and helps in the degradation of damaged/misfolded proteins with the help of the ubiquitin-proteasome system (Hossain et al., 2020). Heat shock proteins bind to the denatured proteins, compelling them to refold into their native conformation and regain their original structure (Feng et al., 2018). In this study, we observed 42 proteins upregulated that belonged to post-translational modification, protein turnover, and chaperones. There was upto 4-fold upregulation of proteasome regulatory particle subunit *Rpt3* (KOG0727), upto 2-fold upregulation of Hsp70 chaperone (*HscA*) (KOG0101), and upto 2.5-fold upregulation of protein geranylgeranyltransferase (KOG1439). The enzyme geranylgeranyltransferase I (GGTase I) aids in the catalysis of the post-translational transfer of lipophilic diterpenoid geranylgeranyl molecule to the cysteine residue of proteins with the termination at CaaX motif (*Rho1p* and *Cdc42p*). This alteration helps in the membrane localization of the protein and thereby rendering it biologically active. *Rho1p* is a regulatory subunit of 1,3-β-D-glucan synthesis and contributes to the cell wall synthesis in fungi which is vital for cell viability under stressful condition of excess metals (Singh et al., 2005).

#### Translation, Ribosomal Structure, and Biogenesis

Different proteins related to protein translation under multimetal stress were overexpressed. Here, we found 60 proteins of translation, ribosomal structure and biogenesis upregulated. There was upto 3.0-fold increase in glutamyl-tRNA synthetase (KOG1147), upto 2.5-fold increase in eukaryotic translation initiation factor 3 subunit B (KOG2314), upto 2.0-fold increase in 60S ribosomal protein L23 (KOG1751), upto 2.3-fold increase in mitochondrial translation initiation factor IF 2 (KOG1144), and 2.0-fold increase 40S ribosomal protein S10b (KOG3344). Similar elements of protein synthesis, such as translation initiation factor 5A, elongation factor 2, 40S and 60S ribosomal proteins, ATP-dependent RNA helicase, and aspartyl-tRNA synthetase, were overexpressed in *Phanerochaete chrysosporium* under Cu stress as a result of the need for production of new proteins or renewal of the damaged proteins (Okay et al., 2020).

#### Intracellular Trafficking and Secretion and Vesicular Transport

Proteins such as ion transporters and other solutes are crucial for processes such as detoxification, cell nutrition, cell signaling, cellular homeostasis, and resistance toward metal stress. These polytopic transmembrane proteins are translated altogether and folded in the endoplasmic reticulum (ER) of the eukaryotic cells that are later ultimately arranged to their respective membrane location through vesicular secretion. During any physiological or stressful environment, transporters undertake several regulated turnovers. Thus, in the process, transporters briefly interact dynamically with multiple proteins (Dimou et al., 2021). In this study, we found 18 proteins of intracellular trafficking and secretion and vesicular transport upregulated. The levels of the proteins were expressed in higher amounts (SEC31) (KOG0307) by 3.3-fold, endosomal cargo receptor (P24) (KOG1692) by 2.9-fold, and mitochondrial inner membrane translocase (KOG2580) by 2.4-fold in *A. fumigatus* under the effect of multimetal stress. The vesicle (Ves) are tissues composed of a lipid bilayer whose size varies ∼nanometers to micrometers. The Ves structures fuse with the plasma membrane of the cell and eject the trapped materials either inside or outside of the cytoplasm. There are three types of intracellular Ves viz. protein complex I (COPI)-coated Ves, protein complex II (COPII)-coated Ves, and BAR-domain protein Ves. These Ves proteins aid in physiological processes, such as the exchange of proteins and RNA intercellularly (Jiang et al., 2020).

#### Energy Production and Conversion

Heavy metals, such as Cd, Cu, Ni, and Zn, function as cofactors in bacteria and fungi. However, excess amounts of these metals are toxic to these cells and also produce reactive oxygen species (Liu et al., 2017). The need for metabolic energy in the fungal increases during abiotic stress, such as exposure to excess heavy metals. Thus, ATPases are responsible for the biochemical and physiological processes by the production of energy. Heavy metal ATPases (HMAs) or P-type ATPases can be categorized into three groups namely, A, B, and C. Further, P-type ATPases are utilized by numerous organisms to facilitate the transport of cations viz. Na^+^, K^+^, and Ca^2+^. To eliminate these excess metals, fungal HMA *Saccharomyces cerevisiae* CCC2 (Group A) localizes metals to metal-containing proteins, for example, in the case of copper metal, copper-containing protein *FET3* in trans-golgi compartment transports metals to the cell membrane *via* efflux pumps, such as cadmium efflux pumps, encoded by fungal HMA *Saccharomyces cerevisiae* PCA1 (Group B and Group C) (Adle et al., 2007). Saitoh et al. (2009) studied the CCC2-type *HMA* gene that targets copper-containing proteins from the fungus *Cochliobolus heterostrophus* by cloning. There was upto 0.78-fold upregulation in the production of V-type ATPase and upto 1-fold upregulation in the production of mitochondrial ATPase subunit ATP4. This gene has other multifarious roles, such as in the formation of dark brown colored melanin pigment located in fungal cell walls, that also sequester metals (Chang et al., 2019).

#### Amino Acid Transport and Metabolism

The nitrogen cycle is essential for nitrogen assimilation and transformation and also for stress tolerance. Heavy metals impact the enzymes that play important role in nitrogen metabolism (Khouja et al., 2014). There was upto 4-fold upregulation in the production of aminotransferase (KOG1549) in response to multimetal. A similar response was observed by Okay et al. (2020), where the production of enzyme aspartate aminotransferase was enhanced in the fungus *Phanerochaete chrysosporium* to tackle the Cu stress. This enzyme has a possible role in the renewal of the mitochondrial NAD/NADH imbalance.

### Analysis of Biological Pathways and Protein–Protein Interactions

In addition to these mechanisms, there are contributions from other regulatory systems, such as cross-talks in various pathways, interconnection amongst these different pathways, and regulation of different genes. These regulatory networks of the microbial proteins are intricate and play crucial roles in resistance to metal contaminants by modifying the series of specific functional proteins/non-proteins and altering the different metabolic enzymes at the cellular level. The metabolic processes related to the detoxification of contaminants are usually regulated by the complete set of proteins and their networks instead of a single enzyme (Zhao and Poh, 2008; Feng et al., 2018).

The STRING analysis displayed protein–protein interaction networks that are related to the resistance and tolerance mechanism *A. fumigatus* PD-18 for multimetals. Protein interactions in the first cluster (green) are involved in ribosome and preinitiation factors (60S and 40S ribosomal proteins). Ribosomal proteins form the protein part of ribosomes and participate in protein synthesis in cells in conjunction with rRNA. Thus, the increased expression of the large subunit of ribosome renders resistance against abiotic stresses, such as heavy metals, radiation, cold, and salt (Liu et al., 2014).

The important hub proteins expressed were cytochrome-c oxidase that is mitochondrial proteins and catalyst in the electron transport chain and is responsible for the transport of heavy metals particularly copper (Dias et al., 2019). The second cluster (blue) had hub protein ATP synthase. The important interconnecting protein in the network is the septin protein of KOG class cell cycle control, cell division, and chromosome partitioning, and is involved in vesicular trafficking and countering the apoptotic cell death initiated by the toxicity of heavy metals. This energy-intensive process is mediated by ATP synthase to maintain the cellular structure and function under the lethal environment of heavy metals. Excess to or above the permissible limit of heavy metals exposure can substantially delimit the growth of organisms. Here, more growth is initiated in the fungal cell as a result of the enhanced activity of ATP synthase (Yıldırım et al., 2011). This was evident in our study by the upregulation of tubulin by 1.1-fold and actin proteins by 2.2-fold which are responsible for cellular division and growth (Marks et al., 1986). This enhanced growth in the fungus also corroborated with our previous study where there was an increased dry weight of the fungal biomass under 30 mg/L multimetal (Dey et al., 2016). These results showed that the proteins in this network played important functions in cell functioning under heavy metals stress.

The mechanism of hexametal uptake by *A. fumigatus* PD-18 has been summarized in Figure 4.

## Conclusion

In this study, we found the fungus *A. fumigatus* PD-18 developed stress coping strategies by secreting a suite of proteins that were either unique or upregulated/overexpressed when compared to the control. The proteomics study revealed that maximum proteins that were upregulated belonged to KOG class translation, ribosomal structure, and biogenesis. This study also highlighted the enhanced expression of antioxidants superoxide dismutase, molecular chaperone heat shock proteins, and involvement of proton transporter, such as ATPase. These proteins are involved in the tolerance and detoxification of multimetals by the fungus. *A. fumigatus* PD-18. Therefore, this investigation on the response of cellular proteomes to multimetal stress enabled us to better understand the cellular mechanism regarding the cumulative effect of the inorganic heavy metals stress on microbes. Further, it will be conducive to screening the key genes coding for enzymes that have higher resistance to these inorganic pollutants with enhanced capability to transform pollutants.

## Data Availability Statement

The mass spectrometry proteomics data have been deposited to the ProteomeXchange Consortium via the PRIDE [1] partner repository with the dataset identifier PXD031741.

## Author Contributions

PD, AM, and NJ contributed to conception and design of the study. PD organized the database and wrote the first draft of the manuscript. PD and S-BH performed the statistical analysis. PD, AM, NJ, DS, and MB wrote sections of the manuscript. All authors contributed to manuscript revision, read, and approved the submitted version.

## Conflict of Interest

PD, S-BH, MB, and NJ were employed by Helmholtz-Centre for Environmental Research-UFZ GmbH. The remaining authors declare that the research was conducted in the absence of any commercial or financial relationships that could be construed as a potential conflict of interest.

## Publisher’s Note

All claims expressed in this article are solely those of the authors and do not necessarily represent those of their affiliated organizations, or those of the publisher, the editors and the reviewers. Any product that may be evaluated in this article, or claim that may be made by its manufacturer, is not guaranteed or endorsed by the publisher.

## Footnotes

1
https://www.prophane.de/

2
https://bioinfogp.cnb.csic.es/tools/venny/

3
https://wolfpsort.hgc.jp/

4
https://string-db.org/
